# Clinical Outcome of Discordant Empirical Therapy and Risk Factors Associated to Treatment Failure in Children Hospitalized for Urinary Tract Infections

**DOI:** 10.3390/children9020128

**Published:** 2022-01-19

**Authors:** Giovanni Autore, Cosimo Neglia, Margherita Di Costanzo, Martina Ceccoli, Gianluca Vergine, Claudio La Scola, Cristina Malaventura, Alice Falcioni, Alessandra Iacono, Antonella Crisafi, Lorenzo Iughetti, Maria Luisa Conte, Luca Pierantoni, Claudia Gatti, Giacomo Biasucci, Susanna Esposito, UTI-Ped-ER Study Group

**Affiliations:** 1 Pediatric Clinic, Department of Medicine and Surgery, University of Parma, 43126 Parma, Italy; giovanniautore@gmail.com (G.A.); negliamino@gmail.com (C.N.); 2 Paediatrics and Neonatology Unit, Guglielmo da Saliceto Hospital, 29122 Piacenza, Italy; m.dicostanzo@ausl.pc.it (M.D.C.); g.biasucci@ausl.pc.it (G.B.); 3 Paediatrics Unit, Department of Medical and Surgical Sciences of Mothers, Children and Adults, University of Modena and Reggio Emilia, 41125 Modena, Italy; martina.ceccoli90@gmail.com (M.C.); lorenzo.iughetti@unimore.it (L.I.); 4 Paediatrics Unit, Rimini Hospital, AUSL Romagna, 47921 Rimini, Italy; gianluca.vergine@auslromagna.it (G.V.); marialuisa.conte@auslromagna.it (M.L.C.); 5 Paediatric Clinic, IRCCS Azienda Ospedaliero-Universitaria di Bologna, 40138 Bologna, Italy; clasc1976@gmail.com; 6 Paediatric Clinic, University of Ferrara, 44124 Ferrara, Italy; mlvcst@unife.it; 7 Paediatric Unit, Forlì Hospital, AUSL Romagna, 47121 Forlì, Italy; alice.falcioni@auslromagna.it; 8 Paediatrics and Neonatology Unit, Ravenna Hospital, AUSL Romagna, 48121 Ravenna, Italy; alessandra.iacono@auslromagna.it; 9 Paediatrics Unit, Santa Maria Nuova Hospital, AUSL-IRCCS of Reggio Emilia, 42123 Reggio Emilia, Italy; antonella.crisafi@ausl.re.it; 10 Paediatric Emergency Unit, IRCCS Azienda Ospedaliero-Universitaria di Bologna, 40138 Bologna, Italy; luca.pierantoni@aosp.bo.it; 11 Paediatric Surgery, University Hospital, 43126 Parma, Italy; clgatti@ao.pr.it

**Keywords:** antibiotic failure, antibiotic resistance, empirical therapy, discordant antibiotic, urinary tract infections

## Abstract

With the spread of antibiotic resistance in pediatric urinary tract infections (UTIs), more patients are likely to be started empirically on antibiotics to which pathogens are later found to be resistant (discordant therapy). However, in-vivo effectiveness may be different from in-vitro susceptibility. Aims of this study were to describe clinical outcomes of discordant empirical treatments in pediatric UTIs and to investigate risk factors associated to treatment failure. This observational, retrospective study was conducted on children hospitalized for febrile UTIs with positive urine culture and started on discordant empirical therapy. Failure rates of discordant treatments and associated risk factors were investigated. A total of 142/1600 (8.9%) patients were treated with inadequate empirical antibiotics. Clinical failure was observed in 67/142 (47.2%) patients, with no fatal events. Higher failure rates were observed for combinations of penicillin and beta-lactamase inhibitors (57.1%). Significant risk factors for failure of discordant treatment were history of recurrent UTIs (95% CI: 1.13–9.98, OR: 3.23, *p* < 0.05), recent use of antibiotics (95% CI: 1.46–21.82, OR: 5.02, *p* < 0.01), infections caused by *Pseudomonas aeruginosa* (95% CI: 1.85–62.10, OR: 7.30, *p* < 0.05), and empirical treatment with combinations of penicillin and beta-lactamase inhibitors (95% CI: 0.94–4.03, OR: 1.94, *p* = 0.05). This study showed that discordant empirical treatments may still be effective in more than half of pediatric UTIs. Clinical effectiveness varies between different discordant antibiotics in pediatric UTIs, and patients presenting risk factors for treatment failure may need a differentiated empirical approach.

## 1. Introduction

Urinary tract infections (UTIs) are among the most common infections in pediatric population, representing a major cause for antibiotic consumption and hospitalization in children [1]. UTIs affect up to 2.8% of children annually in high-income countries, and it is estimated that nearly 8% of females and 2% of males develop at least one episode of UTI within the first eight years of life, with recurrence rates ranging from 8% to 30% [2,3,4,5]. Gram-negative microorganisms of the *Enterobacteriaceae* family are the most common pathogens in pediatric UTIs, with *Escherichia coli* accounting for more than 70% of cases also in recurrent episodes [6,7].

The spread of antibiotic resistance in pediatric UTIs is an increasing public health problem worldwide. Economic burden associated with management of pediatric UTIs resistant to antibiotics is rising, and according to a recent European study, healthcare costs more than doubled for children hospitalized for UTIs caused by resistant pathogens, mainly due to the increased length of stay [8,9]. Resistance rates for common first-line antibiotics, such as amoxicillin and trimethoprim-sulfamethoxazole, already exceed 50% and 25%, respectively [10,11]. These findings prompted the empirical use of broad-spectrum molecules that represent more than one-third of outpatient prescriptions for UTIs [12].

Appropriate empirical treatment is considered the cornerstone of the management of UTIs because untreated infections can lead to serious complications, such as sepsis and renal failure [13]. However, the real effect of inadequate initial antibiotic coverage on long-term sequelae is controversial [14]. Most guidelines suggest a wide range of molecules as equally suitable empirical treatments for UTIs; thus, the choice of initial antibiotic is often based on in-vitro resistance rates reported by surveillance studies [15,16]. Because antibiotic susceptibility testing generally requires 2–3 days from presentation, the need of early treatment to prevent renal damage may result in discordant empirical therapy, defined as initial antibiotic to which infecting isolate is later found to be not susceptible. As resistance rates increase, more patients are likely to be started empirically on discordant therapy. However, in-vivo effectiveness may be different from in-vitro results considering that antibiotic concentrations in urinary tract are often higher than can be achieved in blood [17]. Clinical improvement has been reported in adult patients affected by UTIs despite discordant therapy, questioning the need of using new broad-spectrum antibiotics as first-line treatment [18,19]. However, data on pediatric population are lacking; thus, the real consequences in everyday clinical practice of the increasing antibiotic resistance in pediatric UTIs are still unclear. The aims of this study were to describe clinical outcomes of discordant empirical treatments in children hospitalized for UTIs and to investigate risk factors associated to treatment failure.

## 2. Methods

### 2.1. Study Design and Population

**Figure 1:**
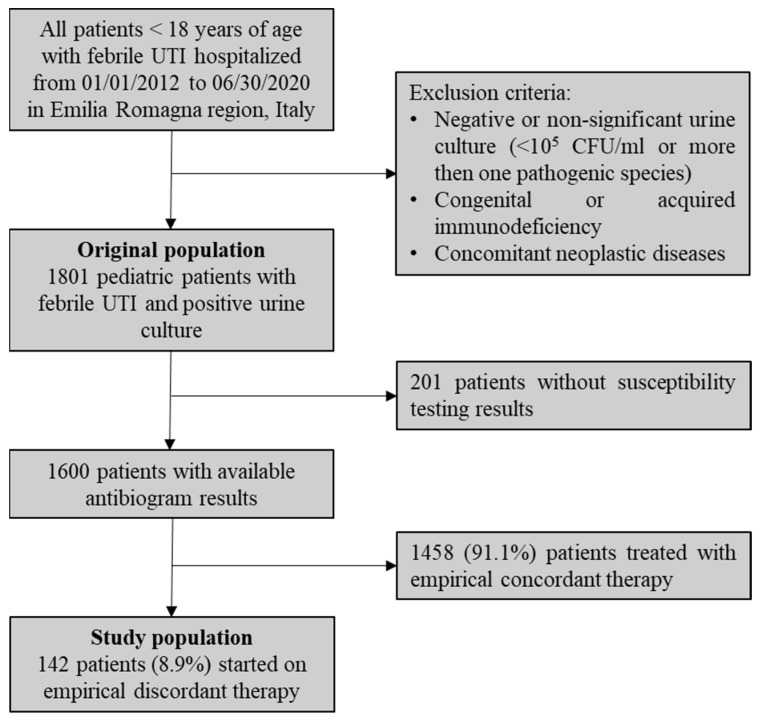
Design of the study, inclusion/exclusion criteria and selection of study population.

This observational, retrospective study was conducted on the population of a previous large multicenter surveillance study that enrolled 1801 children hospitalized for febrile UTIs in pediatric units of Emilia-Romagna Region, Italy, from 1 January 2012 to 30 July 2020 [11]. The original population included hospitalized patients aged under 18 years, presenting with fever and with subsequent positive urine culture, defined as identification of a single pathogen present at ≥10^5^ CFU/mL. Initial exclusion criteria were afebrile patients suggesting lower urinary tract infections, negative or non-significant urine culture, concomitant neoplastic diseases, and congenital or acquired immunodeficiency. All the patients at their first episode and patients with recurrent episodes of UTIs were included. For patients with multiple admissions meeting the inclusion criteria, only the last episode was included. From original population, we further selected only patients with available antibiogram results and empirically treated with discordant therapy, defined as initial antibiotic prescribed at hospital admission to which the pathogen identified in urine culture was proven resistant at susceptibility testing (Figure 1). When combinations of antibiotics were used, the therapy was considered discordant if the isolate tested resistant to both molecules.

### 2.2. Data Evaluation

Medical records were extrapolated and analyzed using a special form for each patient. Data collection was focused on demographic characteristics, medical and urological history, signs and symptoms at onset, blood and urinary laboratory tests, radiological findings (including urinary tract ultrasound, voiding cystourethrography, and renal scintigraphy when performed), any procedures carried out, urine culture and antibiogram results, trends in vital parameters, and antibiotics administered before admission, during hospital stay, and at discharge. Uropathogens were classified according to their antibiotic-resistance patterns. Extended-spectrum beta-lactamase (ESBL)-producing pathogens were defined by direct laboratory test for the presence of ESBLs or phenotypically by proven capacity of hydrolyzing penicillin, first- to third-generation cephalosporins, and aztreonam [20]. According to indications from European Centre for Disease Prevention and Control (ECDC) and the Centers for Disease Control and Prevention (CDC), multidrug-resistant (MDR) pathogens were defined as resistant to at least one molecule in three different antimicrobial categories, and extensively drug-resistant (XDR) pathogens were defined as susceptible to only agents of two or less categories [20]. Resistance patterns not included in ESBL, MDR, or XDR categories were defined as simple resistance.

Treatment failure was defined as persistence of fever, lack of clinical improvement, and need for additional antibiotic therapy after 48 h of empirical treatment. Failure rates were further evaluated according to different antibiotics, uropathogens, and antimicrobial-resistance patterns. Patients were treated in the hospital until they were apyretic, able to feed/eat without vomiting, and in stable clinical conditions for 48 h. All the patients were treated for 10 days, and after discharge, they were treated at home with oral antibiotic therapy. Empiric therapy was changed only in presence of treatment failure according to the clinical definition reported above. Length of stay (LOS) in the hospital and second-line therapies were also analyzed. Risk factors associated to discordant treatment failure were investigated, including clinical, microbiological and laboratory findings, and antibiotic treatments.

### 2.3. Statistical Analysis

Statistical data analyses were performed by using Stata Release 12 (StataCorp, College Station, TX, USA). Descriptive statistics as absolute and relative frequencies, means, and standard deviations (SD) were used to summarize data. Odds ratios (OR) and their corresponding 95% confidence intervals (95% CI) were estimated by univariate logistic regression to assess association between failure of discordant empirical treatment and other variables, such as sex, history of recurrent UTIs antibiotic prophylaxis, antibiotic therapy in previous 30 days, pathogens, and age groups. In the comparison of odds ratios by age group, the age group less than three months was taken as a reference. For odds ratio analysis, an alpha ≤ 0.05 was considered statistically significant.

## 3. Results

From original population of 1801 patients < 18 years of age and hospitalized with febrile UTIs and positive urine culture, susceptibility testing results were available for 1600 (88.9%) patients. As summarized in Figure 1, 142/1600 (8.9%) patients were started empirically on antibiotics to which the corresponding pathogen was later found resistant at antibiogram (discordant therapy). Characteristics of the study population managed empirically with discordant therapy are summarized in Table 1.

*Escherichia coli* was by far the most common uropathogen, accounting for 105 (73.9%) cases, followed by *Klebsiella* spp., *Enterobacter* spp., and *Pseudomonas aeruginosa* in 13 (9.1%), 11 (7.7%), and 7 (4.9%) cases, respectively (Table 2). ESBL-producing pathogens were identified in 24 (16.9%) cases, while 34 (23.9%) infections were caused by MDR or XDR pathogens.

Empirical treatment failed in higher proportion when infections were caused by *Pseudomonas aeruginosa* (85.7%), ESBL-producing pathogens (54.2%), and MDR/XDR microorganisms (58.8%).

The most used empirical treatments were combinations of penicillin and beta-lactamase inhibitors, prescribed in 63 (44.4%) cases, of which amoxicillin/clavulanate was used in 58 (40.8%) patients (Table 3). A total of 99 (69.7%) patients received intravenous antibiotics, while oral route of administration was preferred in 43 (30.3%) cases.

Mean LOS was significantly increased for patients treated with discordant empirical therapies when compared to concordant treatments (6.5 ± 3.3 vs. 5.4 ± 2.7 days, *p*-value < 0.001). Among patients treated with discordant empirical antibiotic, no fatal events were reported, and no one needed transfer to intensive care units.

Overall, discordant empirical treatment failed in 67 patients with a failure rate of 47.2%. Among patients treated with discordant empirical antibiotic, no fatal events were reported, and no one needed transfer to intensive care unit. Among the most used empirical antibiotics, high failure rate was observed for combinations of penicillin and beta-lactamase inhibitors (36/63, 57.1%), while combinations of penicillin and aminoglycosides and third-generation cephalosporins failed less frequently (11/31, 35.5%; 11/28, 39.3%). No statistically significant differences in failure rates were observed between intravenous and oral routes of administration (43.4% vs. 56.4%, *p*-value 0.6).

Table 4 summarizes all the risk factors for failure of discordant empirical treatment that were analyzed. Univariate logistic regression confirmed as significant risk factors for treatment failure history of recurrent UTIs, use of antibiotics during previous 30 days, infections caused by *Pseudomonas aeruginosa*, and empirical treatment with combinations of penicillin and beta-lactamase inhibitor. Simple patterns of antibiotic resistance were associated with an almost-significant protective effect on treatment failure.

## 4. Discussion

This study showed that discordant empirical treatment in pediatric UTIs may be successful in more than half of cases despite proven in-vitro resistance of uropathogens. Independent risk factors for failure of discordant therapy were history of recurrent UTIs, use of antibiotics during previous 30 days, *Pseudomonas aeruginosa* as causative pathogen, and empirical treatment with combinations of penicillin and beta-lactamase inhibitors.

We observed that 8.9% of patients affected by UTI with positive urine culture were empirically treated with discordant antibiotics. This is consistent with previous studies conducted with similar inclusion criteria and not restricted to specific pathogens or antimicrobial-resistance patterns [21]. Among demographic characteristics and features from previous medical history, only history of recurrent UTIs increased the risk of treatment failure in our study. Several previous studies reported that history of recurrent UTIs, antibiotic prophylaxis, and use of antibiotics during previous 30 days are significant risk factors for the development of resistant UTIs in pediatric patients [22,23]. These findings were previously confirmed also in the original population of our epidemiological contest [11]. Recurrent UTIs and recent exposure to antibiotics seem to play a role also in further effectiveness of discordant antibiotics. Patients with these risk factors seem more often infected by uropathogens, presenting multiple virulence factors and higher resistance. Moreover, recurrence of UTIs may in turn be caused by a wide range of urological and non-urological conditions [24].

Predictably, infections caused by *Pseudomonas aeruginosa* had a very high rate of discordant treatment failure due to intrinsic resistance of the pathogen to common empirical antibiotics [25].

Discordance between in-vitro and in-vivo effectiveness varied between different classes of antibiotics, but interestingly, route of administration had no effect on treatment failure rate. This finding implements the emerging evidence that intravenous antibiotic regimens are not required when similar oral alternatives are available. Clinical trials already documented the non-inferiority of entirely oral antibiotic regimens [26,27], and a large Cochrane systematic review confirmed these results [28]. Our study confirms these data and suggests that intravenous route of administration may offer no advantages compared to oral regimens, not even when empirical treatment results are discordant. However, more studies are needed to confirm this finding in populations with different clinical severity.

Combinations of penicillin and beta-lactamase inhibitors showed higher failure rates and were a significant risk factor for treatment failure. On the other hand, discordant third-generation cephalosporins were effective in more than 60% of cases although logistic regression failed to assess a statistically significant protective effect relative to treatment failure. Wang et al. recently reported even better results for discordant third-generation cephalosporins when used in emergency department setting [29]. Among 316 children affected by UTIs caused by ESBL-producing pathogens and inappropriately treated with cephalosporins, they observed clinical improvement in more than 80% of them, and repeated urine cultures sterilized in 65% of cases. A multicenter prospective study conducted on inpatient pediatric UTIs due to ESBL-producing uropathogens reported that discordant use of third-generation cephalosporins had no effect on time to apyrexia and LOS [30]. Our results in this study are in line with those reported by Wang et al. [29], whereas in our original cohort, we already demonstrated that having ESBL or MDR/XDR uropathogens were significantly associated with treatment failure [11].

Reasons beyond different in-vivo performance of equally discordant antibiotic molecules are controversial. A possible explanation is that antibiotics achieve higher concentrations in the urine and renal parenchyma, providing a clinical response despite in-vitro resistance that is often based on blood concentrations [17,31]. The majority of used antibiotics are eliminated in urine and may reach high concentrations in urinary tract. This aspect is probably just a piece of the intricate puzzle of in-vivo interaction between antibiotic molecules and uropathogens. An interesting factor affecting antibiotic resistance in UTIs is the formation of biofilms [32]. It is possible that different antibiotics cause variable effect on pathogens forming biofilms [33]. Moreover, some UTIs may resolve spontaneously regardless of antibiotic effectiveness, similarly to acute otitis media [34].

In this study, LOS in hospital was increased for patients treated with discordant empirical therapy when compared to concordant antibiotics. This is consistent with previous studies reporting the same finding [21]. In fact, discordant empirical therapy shows higher failure rates and may require a subsequent second-line treatment. Conversely, a multicenter prospective study observed no difference in length of stay between patients treated with discordant or concordant therapies [30]. It is possible that variability criteria for discharge may contribute to prolong hospitalization. In fact, some pediatricians may delay a patient’s discharge despite clinical improvement when prescribed treatment results are discordant to susceptibility testing results.

Limitations of this study include its retrospective design and limited representation of some factors in the study population. The lack of follow-up after acute episode precluded the evaluation of effects of discordant empirical treatment on long-term sequelae, such as renal scarring. The definition of treatment failure in the scenario of discordant therapy may underestimate the success rate because some pediatricians may choose to change a therapy despite clinical improvement when it results as discordant to antibiogram results. However, few studies tried to describe the effectiveness of discordant antibiotics in pediatric UTIs. Our study provided a real-life picture of the discordance between in-vitro and in-vivo effectiveness of commonly used antibiotics that is frequently experienced in clinical practice. Defining the risk factors associated to discordant treatment failure, results from this study may help future research on differentiated antibiotic approaches for pediatric UTIs.

The increasing prevalence of uropathogens resistant to commonly used empirical antibiotics in community-acquired pediatric UTIs represents a serious public health problem. The alarming reports of surveillance studies may prompt the use of antibiotics with ever broader spectrum of action as empirical therapy. However, it is well known that inappropriate antibiotic use leads to increased antibiotic resistance in community-acquired UTIs, as demonstrated by higher resistance rates reported in countries where antibiotics are available over the counter [35]. Moreover, changing antibiotic prescribing patterns often facilitate the emergence of new resistances, as demonstrated in regions where use of amoxicillin/clavulanate or third-generation cephalosporins increased [11,36]. To avoid this vicious cycle, it is important not only to constantly monitor the prevalence of antibiotic resistance and to promote diversified empirical treatments based on risk factors for resistant infections but also to investigate the in-vivo effectiveness of empirical antibiotics already in use and its discordance with in-vitro findings.

## 5. Conclusions

This study showed that discordant empirical antibiotics may still be effective in more than half of children hospitalized for UTI, questioning the need to broaden the spectrum of action of empirical treatment. Clinical effectiveness of recommended empirical antibiotics may often overcome the increasing in-vitro resistance reported by surveillance studies. History of recurrent UTIs, use of antibiotic during 30 days prior to admission, infections caused by *Pseudomonas aeruginosa*, and use of combinations of penicillin and beta-lactamase inhibitors are the main risk factors for failure of discordant treatment. In this groups of patients, a differentiated empirical approach is needed, and the second-line therapy should be chosen on the basis of antibiogram’s results.

## Figures and Tables

**Table 1:** Characteristics of the study population managed empirically with discordant therapy and clinical, laboratory, and radiological features at admission.

Characteristic	*N* = 142
Mean age, years (SD)	1.8 (3.1)
Sex, *n*	
Males	85 (59.9%)
Females	57 (40.1%)
Prenatal pyelectasis, *n*	15 (10.6%)
Prematurity at birth, *n*	27 (19.0%)
Urological malformations, *n*	23 (16.2%)
VUR, *n*	13 (9.1%)
History of recurrent UTIs, *n*	23 (16.2%)
Antibiotic prophylaxis, *n*	13 (9.1%)
Antibiotic therapy in previous 30 days, *n*	19 (13.4%)
Pyelectasis, *n*	49 (34.5%)
Mean CRP, mg/dL (SD)	7.4 (8.3)
Mean WBC count, cell/mm^3^ (SD)	15,257.3 (5828.0)
Mean treatment delay from fever onset, days (SD)	1.6 (1.5)

CRP, C-reactive protein; UTI, urinary tract infection; VUR, vesicoureteral reflux; WBC, white blood cell; SD, standard deviation.

**Table 2:** Prevalence of different uropathogens and antibiotic-resistance patterns and associated rates of treatment failure.

Pathogens	*n* (%)	Treatment Failure *n* (%)
*Escherichia coli*	105 (73.9)	48 (45.7)
*Klebsiella* spp.	13 (9.1)	7 (53.8)
*Enterobacter* spp.	11 (7.7)	4 (36.4)
*Pseudomonas aeruginosa*	7 (4.9)	6 (85.7)
*Enterococcus faecalis*	2 (1.4)	1 (50.0)
*Proteus mirabilis*	2 (1.4)	1 (50.0)
*Citrobacter* spp.	2 (1.4)	-
ESBL	24 (16.9)	13 (54.2)
MDR/XDR	34 (23.9)	20 (58.8)
Simple resistance	105 (73.9)	45 (42.8)

ESBL, extended-spectrum beta-lactamase-producing; MDR, multidrug-resistant; XDR, extensively drug-resistant.

**Table 3:** Discordant empirical antibiotics and associated failure rates.

Empirical Therapy	*n* (%)	Treatment Failure (%)
Penicillin/beta-lactamase inhibitor combinations	63 (44.4)	36 (57.1)
Penicillin/aminoglycoside combinations	31 (21.8)	11 (35.5)
3rd-generation cephalosporins	28 (19.7)	11 (39.3)
Penicillins	11 (7.7)	4 (36.4)
Cephalosporin/aminoglycoside combinations	3 (2.1)	1 (33.3)
Aminoglycosides	1 (0.7)	-
2nd-generation cephalosporins	1 (0.7)	1 (100)
Fluoroquinolones	1 (0.7)	1 (100)
Other	3 (2.1)	-

**Table 4:** Univariate logistic regression analysis of risk factors for failure of discordant empirical treatment.

Parameter	Odds Ratio (OR)	95 % CI	*p* Value
Male	0.69	0.35–1.36	0.29
Age groups			
<3 months	1.00		
3 months–2 years	1.94	0.89–4.20	0.09
2–6 years	1.76	0.53–5.88	0.35
>6 years	2.20	0.73–6.65	0.16
History of recurrent UTIs	3.23	1.13–9.98	<0.05
VUR	1.67	0.42–7.44	0.42
Urological malformations	1.98	0.55–7.97	0.23
Pyelectasis	1.54	0.69–3.45	0.25
Antibiotic prophylaxis	1.10	0.28–4.58	0.88
Antibiotic therapy in previous 30 days	5.02	1.46–21.82	<0.01
ESBL	1.36	0.51–3.70	0.49
MDR/XDR	1.85	0.79–4.40	0.12
Simple resistance pattern	0.51	0.22–1.16	0.08
*Escherichia coli*	0.80	0.35–1.81	0.55
*Pseudomonas aeruginosa*	7.30	1.85–62.10	<0.05
*Klebsiella* spp.	1.34	0.36–5.10	0.61
*Enterobacter* spp.	0.62	0.13–2.57	0.45
Discordant treatment with penicillin/beta-lactamase inhibitor combinations	1.94	0.94–4.03	0.05
Discordant treatment with 3rd-generation cephalosporins	0.80	0.32–2.00	0.61
Discordant treatment with penicillins + aminoglycoside	0.56	0.23–1.34	0.15
Intravenous route of administration	0.59	0.26–1.34	0.17

## Data Availability

All data are included in the manuscript.

## References

[1] Spencer J.D., Schwaderer A., McHugh K., Hains D.S. (2010). Pediatric urinary tract infections: An analysis of hospitalizations, charges, and costs in the USA. Pediatr Nephrol..

[2] Freedman A.L. (2005). Urologic Diseases in America Project. Urologic diseases in North America Project: Trends in resource utilization for urinary tract infections in children. J. Urol..

[3] Montini G., Tullus K., Hewitt I. (2011). Febrile urinary tract infections in children. N. Engl. J. Med..

[4] Keren R., Shaikh N., Pohl H., Gravens-Mueller L., Ivanova A., Zaoutis L., Patel M., Deberardinis R., Parker A., Bhatnagar S. (2015). Risk Factors for Recurrent Urinary Tract Infection and Renal Scarring. Pediatrics.

[5] Copp H.L., Halpern M.S., Maldonado Y., Shortliffe L.D. (2011). Trends in hospitalization for pediatric pyelonephritis: A population based study of California from 1985 to 2006. J. Urol..

[6] Saperston K.N., Shapiro D.J., Hersh A.L., Copp H.L. (2014). A Comparison of Inpatient vs. Outpatient Resistance Patterns of Pediatric Urinary Tract Infection. J. Urol..

[7] Sakran W., Smolkin V., Odetalla A., Halevy R., Koren A. (2015). Community-acquired urinary tract infection in hospitalized children: Etiology and antimicrobial resistance. A comparison between first episode and recurrent infection. Clin. Pediatr..

[8] Sood A., Penna F.J., Eleswarapu S., Pucheril D., Weaver J., Abd-El-Barr A.-E., Wagner J.C., Lakshmanan Y., Menon M., Trinh Q.-D. (2015). Incidence, admission rates, and economic burden of pediatric emergency department visits for urinary tract infection: Data from the nationwide emergency department sample, 2006 to 2011. J. Pediatr. Urol..

[9] Nieminen O., Korppi M., Helminen M. (2017). Healthcare costs doubled when children had urinary tract infections caused by extended-spectrum β-lactamase-producing bacteria. Acta. Paediatr..

[10] Bryce A., Costelloe C., Wootton M., Butler C.C., Hay A.D. (2018). Comparison of risk factors for, and prevalence of, antibiotic resistance in contaminating and pathogenic urinary Escherichia coli in children in primary care: Prospective cohort study. J. Antimicrob. Chemother..

[11] Esposito S., Maglietta G., Di Costanzo M., Ceccoli M., Vergine G., La Scola C., Malaventura C., Falcioni A., Iacono A., Crisafi A. (2021). Retrospective 8-Year Study on the Antibiotic Resistance of Uropathogens in Children Hospitalised for Urinary Tract Infection in the Emilia-Romagna Region, Italy. Antibiotics.

[12] Copp H.L., Shapiro D.J., Hersh A.L. (2011). National ambulatory antibiotic prescribing patterns for pediatric urinary tract infection, 1998–2007. Pediatrics.

[13] Hewitt I.K., Zucchetta P., Rigon L., Maschio F., Molinari P.P., Tomasi L., Toffolo A., Pavanello L., Crivellaro C., Bellato S. (2008). Early treatment of acute pyelonephritis in children fails to reduce renal scarring: Data from the Italian Renal Infection Study Trials. Pediatrics.

[14] Shaikh N., Mattoo T.K., Keren R., Ivanova A., Cui G., Moxey-Mims M., Majd M., Ziessman H.A., Hoberman A. (2016). Early Antibiotic Treatment for Pediatric Febrile Urinary Tract Infection and Renal Scarring. JAMA Pediatr..

[15] American Academy of Pediatrics (2011). Subcommittee on Urinary Tract Infection, Steering Committee on Quality Improvement and Management Urinary Tract Infection: Clinical Practice Guideline for the Diagnosis and Management of the Initial UTI in Febrile Infants and Children 2 to 24 Months. Pediatrics.

[16] National Institute for Health and Care Excellence Urinary Tract Infection in under 16s: Diagnosis and Management. https://www.nice.org.uk/guidance/cg54.

[17] Frimodt-Møller N. (2002). Correlation between pharmacokinetic/pharmacodynamic parameters and efficacy for antibiotics in the treatment of urinary tract infection. Int. J. Antimicrob. Agents.

[18] Mark D.G., Hung Y.-Y., Salim Z., Tarlton N.J., Torres E., Frazee B.W. (2021). Third-Generation Cephalosporin Resistance and Associated Discordant Antibiotic Treatment in Emergency Department Febrile Urinary Tract Infections. Ann. Emerg. Med..

[19] Frazee B.W., Trivedi T., Montgomery M., Petrovic D.F., Yamaji R., Riley L. (2018). Emergency Department Urinary Tract Infections Caused by Extended-Spectrum beta-Lactamase-Producing Enterobacteriaceae: Many Patients Have No Identifiable Risk Factor and Discordant Empiric Therapy Is Common. Ann. Emerg. Med..

[20] Magiorakos A.P., Srinivasan A., Carey R.B., Carmeli Y., Falagas M.E., Giske C.G., Harbarth S., Hindler J.F., Kahlmeter G., Olsson-Liljequist B. (2012). Multidrug-Resistant, Extensively Drug-Resistant and Pandrug-Resistant Bacteria: An International Expert Proposal for Interim Standard Definitions for Acquired Resistance. Clin. Microbiol. Infect..

[21] Jerardi K.E., Auger K.A., Shah S.S., Hall M., Hain P.D., Myers A.L., Williams D.J., Tieder J.S. (2012). Discordant antibiotic therapy and length of stay in children hospitalized for urinary tract infection. J. Hosp. Med..

[22] Zhu F.H., Rodado M.P., Asmar B.I., Salimnia H., Thomas R., Abdel-Haq N. (2019). Risk factors for community acquired urinary tract infections caused by extended spectrum β-lactamase (ESBL) producing Escherichia coli in children: A case control study. Infect. Dis..

[23] Selekman R.E., Shapiro D.J., Boscardin J., Williams G., Craig J.C., Brandström P., Pennesi M., Roussey-Kesler G., Hari P., Copp H.L. (2018). Uropathogen Resistance and Antibiotic Prophylaxis: A Meta-analysis. Pediatrics.

[24] Stein R., Dogan H.S., Hoebeke P., Kočvara R., Nijman R.J., Radmayr C., Tekgül S. (2015). European Association of Urology; European Society for Pediatric Urology. Urinary tract infections in children: EAU/ESPU guidelines. Eur. Urol..

[25] Bitsori M., Maraki S., Koukouraki S., Galanakis E. (2012). Pseudomonas aeruginosa urinary tract infection in children: Risk factors and outcomes. J. Urol..

[26] Montini G., Toffolo A., Zucchetta P., Dall’Amico R., Gobber D., Calderan A., Maschio F., Pavanello L., Molinari P.P., Scorrano D. (2007). Antibiotic treatment for pyelonephritis in children: Multicentre randomised controlled non-inferiority trial. BMJ.

[27] Neuhaus T.J., Berger C., Buechner K., Parvex P., Bischoff G., Goetschel P., Husarik D., Willi U., Molinari L., Rudin C. (2008). Randomised trial of oral versus sequential intravenous/oral ephalosporins in children with pyelonephritis. Eur. J. Pediatr..

[28] Strohmeier Y., Hodson E.M., Willis N.S., Webster A., Craig J. (2014). Antibiotics for acute pyelonephritis in children. Cochrane Database Syst. Rev..

[29] Wang M.E., Lee V., Greenhow T.L., Beck J., Bendel-Stenzel M., Hames N., McDaniel C., King E., Sherry W., Parmar D. (2020). Clinical Response to Discordant Therapy in Third-Generation Cephalosporin-Resistant UTIs. Pediatrics.

[30] Madhi F., Jung C., Timsit S., Levy C., Biscardi S., Lorrot M., Grimprel E., Hees L., Craiu I., Galerne A. (2018). Urinary-tract Infection due to Extended-Spectrum Beta-lactamase–producing Enterobacteriaceae in Children Group. Febrile urinary-tract infection due to extended-spectrum beta-lactamase-producing Enterobacteriaceae in children: A French prospective multicenter study. PLoS ONE.

[31] Chastain D.B., King S.T., Stover K.R. (2018). Rethinking urinary antibiotic breakpoints: Analysis of urinary antibiotic concentrations to treat multidrug resistant organisms. BMC Res. Notes.

[32] Zhao F., Yang H., Bi D., Khaledi A., Qiao M. (2020). A systematic review and meta-analysis of antibiotic resistance patterns, and the correlation between biofilm formation with virulence factors in uropathogenic *E. coli* isolated from urinary tract infections. Microb. Pathog..

[33] González M.J., Robino L., Iribarnegaray V., Zunino P., Scavone P. (2017). Effect of different antibiotics on biofilm produced by uropathogenic Escherichia coli isolated from children with urinary tract infection. Pathog. Dis..

[34] Venekamp R.P., Sanders S.L., Glasziou P.P., Del Mar C.B., Rovers M.M. (2015). Antibiotics for acute otitis media in children. Cochrane Database Syst. Rev..

[35] Bryce A., Hay A.D., Lane I.F., Thornton H.V., Wootton M., Costelloe C. (2016). Global prevalence of antibiotic resistance in paediatric urinary tract infections caused by Escherichia coli and association with routine use of antibiotics in primary care: Systematic review and meta-analysis. BMJ.

[36] Park J., Kang H., Kwak E., Rhim J.-W., Ahn Y., Lee H., Jeong D., Kang J. (2020). Impact of Antibiotic Prescribing Patterns on Susceptibilities of Uropathogens in Children below 24 Months Old. Antibiotics.

